# Protective effects of pentoxifylline against chlorine-induced acute lung injury in rats

**DOI:** 10.1186/s40360-023-00645-2

**Published:** 2023-02-27

**Authors:** Meng-meng Liu, Jiang-zheng Liu, Chen-qian Zhao, Peng Guo, Zhao Wang, Hao Wu, Weihua Yu, Rui Liu, Chun-xu Hai, Xiao-di Zhang

**Affiliations:** 1 Department of Health Service, Logistics College of Chinese People’s Armed Police Force, Tianjin, 300309 China; 2 Department of Toxicology, Shaanxi Key Lab of Free Radical Biology and Medicine, the Ministry of Education Key Lab of Hazard Assessment and Control in Special Operational Environment, School of Public Health, Fourth Military Medical University, Xi’an, 710032 China

**Keywords:** Chlorine, Pentoxifylline, Oxidative stress, Hypoxia, Autophagy

## Abstract

**Objective:**

Chlorine is a chemical threat agent that can be harmful to humans. Inhalation of high levels of chlorine can lead to acute lung injury (ALI). Currently, there is no satisfactory treatment, and effective antidote is urgently needed. Pentoxifylline (PTX), a methylxanthine derivative and nonspecific phosphodiesterase inhibitor, is widely used for the treatment of vascular disorders. The present study was aimed to investigate the inhibitory effects of PTX on chlorine-induced ALI in rats.

**Methods:**

Adult male Sprague-Dawley rats were exposed to 400 ppm Cl_2_ for 5 min. The histopathological examination was carried out and intracellular reactive oxygen species (ROS) levels were measured by the confocal laser scanning system. Subsequently, to evaluate the effect of PTX, a dose of 100 mg/kg was administered. The activities of superoxide dismutase (SOD) and the contents of malondialdehyde (MDA), glutathione (GSH), oxidized glutathione (GSSG) and lactate dehydrogenase (LDH) were determined by using commercial kits according to the manufacturer’s instructions. Western blot assay was used to detect the protein expressions of SOD1, SOD2, catalase (CAT), hypoxia-inducible factor (HIF)-1α, vascular endothelial growth factor (VEGF), occludin, E-cadherin, bcl-xl, LC 3, Beclin 1, PTEN-induced putative kinase 1 (PINK 1) and Parkin.

**Results:**

The histopathological examination demonstrated that chlorine could destroy the lung structure with hemorrhage, alveolar collapse, and inflammatory infiltration. ROS accumulation was significantly higher in the lungs of rats suffering from inhaling chlorine (*P*<0.05). PTX markedly reduced concentrations of MAD and GSSG, while increased GSH (*P*<0.05). The protein expression levels of SOD1 and CAT also decreased (*P*<0.05). Furthermore, the activity of LDH in rats treated with PTX was significantly decreased compared to those of non-treated group (*P*<0.05). Additionally, the results also showed that PTX exerted an inhibition effect on protein expressions of HIF-1α, VEGF and occludin, and increased the level of E-cadherin (*P*<0.05). While the up-regulation of Beclin 1, LC 3II/I, Bcl-xl, and Parkin both in the lung tissues and mitochondria, were found in PTX treated rats (*P*<0.05). The other protein levels were decreased when treated with PTX (*P*<0.05).

**Conclusion:**

PTX could ameliorate chlorine-induced lung injury via inhibition effects on oxidative stress, hypoxia and autophagy, thus suggesting that PTX could serve as a potential therapeutic approach for ALI.

**Supplementary Information:**

The online version contains supplementary material available at 10.1186/s40360-023-00645-2.

## Introduction

Chlorine, as a respiratory irritant, is widely used in numerous industrial processes, such as plastics, synthetic fibers, dyes, pesticides, and pharmaceutical manufacturing [1–3]. Injuries due to chlorine exposure are usually the result of accidents at swimming pools and the mixing of household agents [4]. Moreover, as a traditional chemical weapon, chlorine is still considered a terrorist threat [5–7]. In World War I, German troops released more than 150 tons of chlorine on April 22, 1915, in Ieper of Belgium. This attack killed up to 5000 and caused injuries on both sides [8]. No matter accidental or deliberate, the release of chlorine poses a significant threat to public health [9–11]. Low concentration chlorine acts as an eye and oral mucous membrane irritant [12], but at the high level, it may induce damage to the lung, even resulting in acute lung injury (ALI) and acute respiratory distress syndrome (ARDS). Although there have been a number of therapeutic interventions recognized over the past couple of years, there is still no specific antidote against chlorine poisoning [13]. Searching for novel drugs remains urgently.

Reactive oxygen (ROS) is known to contribute to the pathogenesis of ALI/ARDS, which may cause the endothelial and epithelial barrier dysfunctions [14, 15]. Through upregulating the expression of adhesion molecules, ROS may amplify the tissue damage and pulmonary edema. In a rats model of LPS-induced ALI, Duan et al. found that inhibited ROS might decrease the expression of adhesion molecules (ICAM-1 and VCAM-1), then attenuated ALI [16].

Hypoxemia is one of the main features of ALI, which is predominantly governed by hypoxia-inducible factor (HIF) [17]. HIF-1 is an oxygen-dependent transcriptional activator that is widely expressed in tissue during hypoxia [18]. HIF-1α, the oxygen-regulated subunit of HIF-1, has been identified to play an important pathophysiological role in maintaining oxygen homeostasis. Under normal conditions, HIF-1α is degraded by ubiquitin-dependent proteasomal. When under hypoxia, the HIF-1α subunit is stabilized and accumulates in the nucleus, and then regulates diverse processes [19]. Moreover, HIF-1 binds to hypoxia-response elements and initiates transcription of various hypoxia-adaptive genes, such as vascular endothelial growth factor (VEGF). As the most potent endothelial specific mitogen, VEGF recruits endothelial cell into hypoxic foci to regulate its function [20]. Our group previously reported that HIF-1α and VEGF levels increased in rat lung tissue after phosgene exposure [21, 22]. Li et al. showed that emodin alleviated pulmonary inflammation in rats with LPS-induced ALI by inhibiting the mTOR/HIF-1α/VEGF signaling pathway [23]. E-cadherin is a transmembrane glycoprotein which presents on the lateral surfaces of epithelial cells and functions as a cell adhesion [24]. Loss of E-cadherin is associated with lung diseases such as asthma and chronic obstructive pulmonary disease [25]. HIF-1α signaling pathway may play a key role in the development of ALI. However, the effects of PTX on hypoxia signaling pathway still need to be explored.

Autophagy is a process of cell self-renewal that is dependent on the degradation of the cytoplasmic proteins or organelles of lysosomes [26]. Extensive work has been performed to confirm that autophagy is involved in the occurrence and development of ALI [27]. In the early stages (1 h and 2 h) of ALI induced by LPS, autophagy reached a peak at 2 h. As the ALI process progressed, autophagy decreased in a time-dependently manner [28]. The role of autophagy in ALI is still unclear. In cecal ligation and puncture (CLP)-induced septic mice, the emergence of autophagy alleviated the cytokine excessive release and lung injury, describing a protective role [29]. In vivo, autophagy aggravated oxidative stress in alveolar epithelial cells in H9N2 influenza virus infection [30]. However, there is no direct evidence for the effect of autophagy on chlorine-induced ALI.

Historically, the nonselective phosphodiesterase inhibitor pentoxifylline (PTX) is reported for clinical use since 1972 and the LD50 oral dosage in rats is 1170 mg/kg. Because there appears no serious drug–drug interaction, PTX can be used easily in vascular disease, including peripheral vascular disease, cerebrovascular disease, and a number of other conditions involving a defective regional microcirculation [31]. These beneficial effects are thought to be due to its anti-inflammatory properties by inhibiting the production of tumor necrosis factors [32]. More importantly, it is widely reported that PTX has been shown to inhibit liver ischemia/reperfusion injury, abdominal compartment syndrome, and intermittent hypobaric hypoxia in experimental animals due to its antioxidant function [33, 34]. Recent research also implied therapeutic effects of PTX on a model of acid-induced ALI and endotoxin-induced ALI [35–38]. Furthermore, Mostafa-Hedeab et al. reported that PTX might exert a protective effects in COVID-19 [39]. While, as an effective drug candidate in the treatment of ALI induced by chlorine, it still needs to be explored.

Thus, this study was aimed to investigate the potential effects of PTX on ALI induced by chlorine. Here, the status of oxidative stress, hypoxia, as well as autophagy in lung tissues were analyzed. The characteristics of “New use of old drugs” can be reflected on PTX.

## Materials and methods

### Chemicals and reagents

Chlorine was obtained from Jinghua Gas Co., Ltd. (Changzhou, China). Pentoxifylline was provided by Sigma (St. Louis, MO). Kits for detecting the activity of lactate dehydrogenase (LDH), superoxide dismutase (SOD), malondialdehyde (MDA), glutathione (GSH) and oxidized glutathione (GSSG) were supplied by Nanjing Jiancheng Bio-Engineering Institute Co., Ltd. Primary antibodies against VEGF, PTEN-induced putative kinase 1 (PINK1), Parkin and cytochrome-c oxidase subunit IV (COX IV) were brought from Santa Cruz Biotechnology (Santa Cruz, CA). Antibodies against occludin and E-cadherin were bought from Abcam (Cambridge, MA, USA). Antibodies against SOD 2 and Beclin 1 were bought from CUSABIO BIOTECH CO., Ltd. (Wuhan, China). Antibodies against HIF-1α, catalase (CAT), bcl-xl and β-actin were bought from Merck Millipore Technology (Burlington, MA), Proteintech Co., Ltd., (Wuhan, China), Cell Signaling Technology (Boston, USA) and Sigma (St. Louis, MO) respectively. Dihydroethidium (DHE) was purchased from Beyotime Co., Ltd. (Shanghai, China).

### Animals and experimental design

Adult male Sprague-Dawley rats (4–6 weeks old, weighing 200–220 g) were provided by the Experimental Animal Center of the Fourth Military Medical University. The animals were housed in cages (6 rats per cage) under a permanent temperature of 20–25 °C and a 12 h light/dark cycle. All the rats were allowed free access to food and water. Efforts were made to minimize animal suffering. In this study, animal model of ALI was induced by inhaling chlorine (400 ppm) for 5 min. After validation of ALI model, according to a random number table, the rats were assigned to four experimental groups (6 rats/group). (1) normal control (NC) group; (2) chlorine group;(3) chlorine + PTX group; (4) PTX group. In addition, rats in the PTX and chlorine +PTX groups were intragastrically administrated with PTX (100 mg/kg) 30 min before chlorine exposure and treatment 15 min after chlorine exposure. The NC and chlorine-treated groups were orally administered with equal amounts of normal saline at the same time.

### Histologic examination

The middle right lung lobes of the rats were fixed in 4% formaldehyde for 24 h. After dehydrated, the sections were embedded in paraffin and sliced at 3 μm. Following deparaffinized and dehydrated, the sectioned tissues were stained with hematoxylin (5 min) and eosin (1–2 min) (H&E). A light microscope (BX51; Olympus Corporation, Japan) was used to observe the extent of histological lung injury.

### Detection of ROS formation

According to the previously described method [38], the intracellular ROS level was detected using the fluorescent dye DHE. Then, the tissue was collected and incubated for 30 min at 37 °C in the dark with 10 μM DHE and 10 μM Hoechst. After washed 3 times with PBS, the tissues were immediately observed by a laser scanning confocal microscopy (FV10i; Olympus Corporation, Japan).

### Preparation of bronchoalveolar lavage fluid (BALF)

The BALF in the lungs was collected as per Liu et al. [40]. In brief, rats were euthanized with intraperitoneal pentobarbital sodium, then the bronchus and lung were exposed. A 3-mm endotracheal cannula was inserted into their trachea. After ligating the hilum of right lung, the left lung was lavaged with 5 mL ice-cold normal saline, which was which retrieved, and the recovery rate was > 90%. The BALF samples were centrifuged (2000 r/min and 4 °C for 10 min) to pellet the cells. Supernatants were removed and stored at − 80 °C.

### Determination of LDH

A commercial kit was used to determine the amount of LDH release following the manufacturer’s protocol. Briefly, the samples were transferred to 96-well plates and incubated at 37 °C for 15 min in the presence of 1 mg/ml NADH. Then 2,4-dinitrophenylhydrazine was added to the samples at 37 °C for another 15 min. The reaction was stopped by addition of 0.4 M NaOH. Data was determined as the absorbance at 450 nm using a spectrophotometric microplate reader.

### Determination of levels of MDA, SOD, GSH, and GSSG

The contents of MDA, SOD, GSH and GSSG in serum were determined according to the Kit commercial instructions.

### Western blotting

The experimental procedure of Western blot analysis was carried out as Guo et al. [41]. Lung tissues were stored at − 80 °C immediately after rats were sacrificed. Tissue samples (100 mg) were ground with a homogenizer in 1 mL of RIPA lysis buffer with 1 mM PMSF and protease inhibitor. Then the homogenate was centrifuged for 20 min at 14400 r/min at 4 °C to collect supernatant. A bicinchoninic acid (BCA) assay (Thermo Scientifc, MA, USA) was applied to determine the protein concentration. After mixed with loading buffer, the supernatants were heated at 100 °C for 10 min at a ratio of 1:1. Equal amounts of the total proteins from each sample were separated by 6–15% SDS-PAGE and transferred onto polyvinylidene difluoride membranes (PVDF; EMD Millipore, Burlington, MA, USA). After blocked with 5% skim milk for 2 h, the blotted membranes were washed with 0.1% Tween-TBS (TBST), and subsequently incubated with the primary antibodies at 4 °C overnight. Then the membranes were washed with TBST buffer three times and incubated with the corresponding secondary antibodies at room temperature for 1 h. After washed with TBST again, the bands were visualized by an enhanced chemiluminescent (ECL) reagent (Thermo Scientifc, MA, USA).

### Statistical analysis

All data were expressed as mean ± standard deviation (SD) and analyzed with a one-way analysis of variance (ANOVA) followed by Tukey’s post hoc test. All the analyses were assessed using the SPSS 13.0. *P* < 0.05 was considered statistically significant.

## Results

### Effect of chlorine on the histological changes and ROS accumulation

**Fig. 1:**
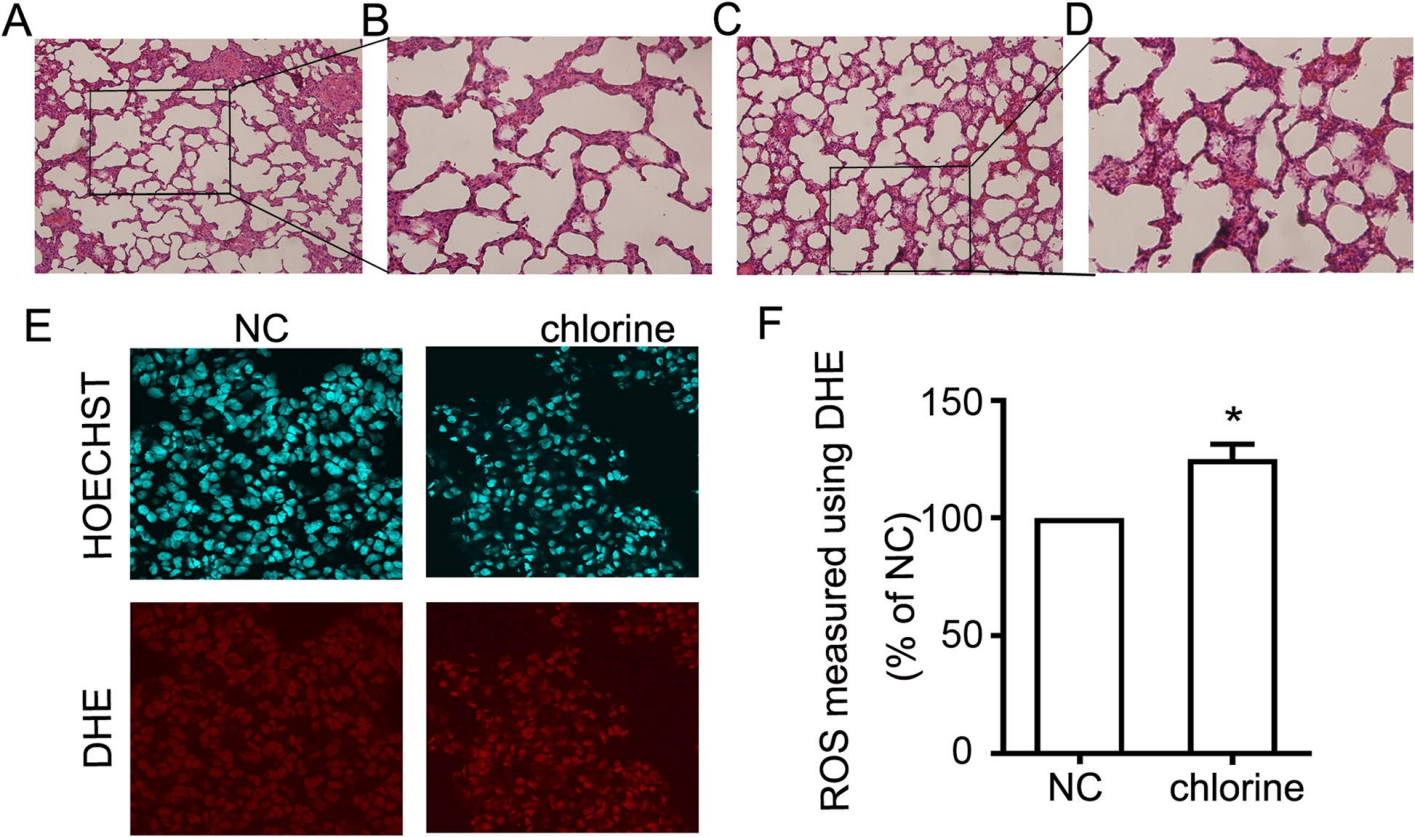
Pathologic changes and ROS accumulation in rats exposed to chlorine. **A** H&E staining in the lungs of rats in NC group (× 200); **B** H&E staining in the lungs of rats in NC group (× 400); **C** H&E staining in the lungs of rats in chlorine group (× 200); **D** H&E staining in the lungs of rats in chlorine group (× 400); **E** Confocal microscopy of the lung tissue (× 600); **F** ROS production measured using DHE. Data are presented as mean ± S.D. (*n* = 3). *P < 0.05 compared with the normal group. H&E: hematoxylin and eosin; NC: normal control; DHE: Dihydroethidium

First, H&E staining was performed to observe the abnormalities of gross features in the lungs after chlorine exposure under a light microscope. As shown in Fig. 1A-D, the rats in the NC group displayed normal appearance and no other histological alteration was observed. In contrast, the lung tissues collected from the group expose to chlorine exhibited marked histopathologic changes, such as alveolar wall thinness, edema, hemorrhage and interstitial infiltration by neutrophils. The airway pathology led to abnormalities in the lung parenchyma with alternating areas of emphysema and atelectasis. Thus, the ALI model had been successfully constructed. Applying this model, the ROS accumulation was measured by DHE. This probe was oxidized to form intermediate probe-derived radicals that were successively oxidized to generate the corresponding fluorescent products [17]. The results demonstrated that ROS level was significantly increased in chlorine-treated group (*P*<0.05) (Fig. 1E-F).

### Effects of PTX on levels of MDA, GSH, GSSG and SOD

**Fig. 2:**
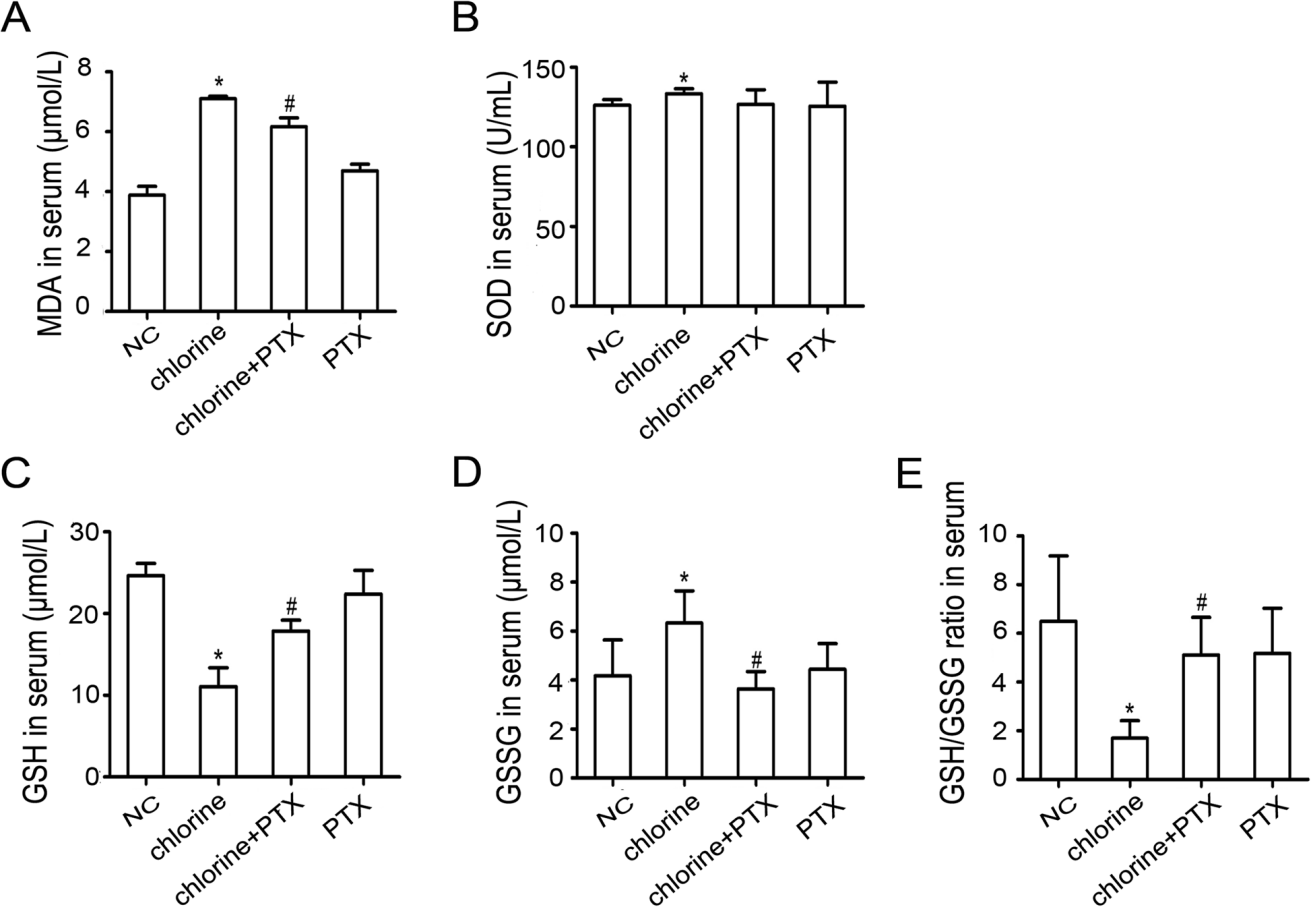
The effect of PTX on the content of MDA, SOD, GSH, GSSG and GSH/GSSG ratio. **A** The content of MDA, **B** the level of SOD, **C** the content of GSH, **D** the content of GSSG and **E** GSH/GSSG ratio. * *P* < 0.05 compared with the normal group. ^#^*P* < 0.05 compared with chlorine-treated group. NC: normal control; MDA: malondialdehyde; SOD: superoxide dismutase; GSH: Glutathione; GSSG: oxidized glutathione; PTX: pentoxifylline

To investigate the effects of PTX on oxidative stress, the expressions of biomarkers for oxidative stress, such as MDA, GSH, GSSG and SOD were detected by commercial assay kits. It was confirmed that the levels of MDA, SOD and GSSG in chlorine treated rats were up-regulated (*P*<0.05) when compared with the NC group, while the effects of PTX administration were pronounced (*P*<0.05), except SOD activity. Moreover, expose to chlorine decreased the levels of GSH and GSH/GSSG ratio (*P*<0.05). Administration of PTX to animals remarkably up-regulated these indexes as compared with rats in the model group (*P*<0.05) (Fig. 2).

### Changes in the protein expression levels of SOD1, SOD2 and CAT

**Fig. 3:**
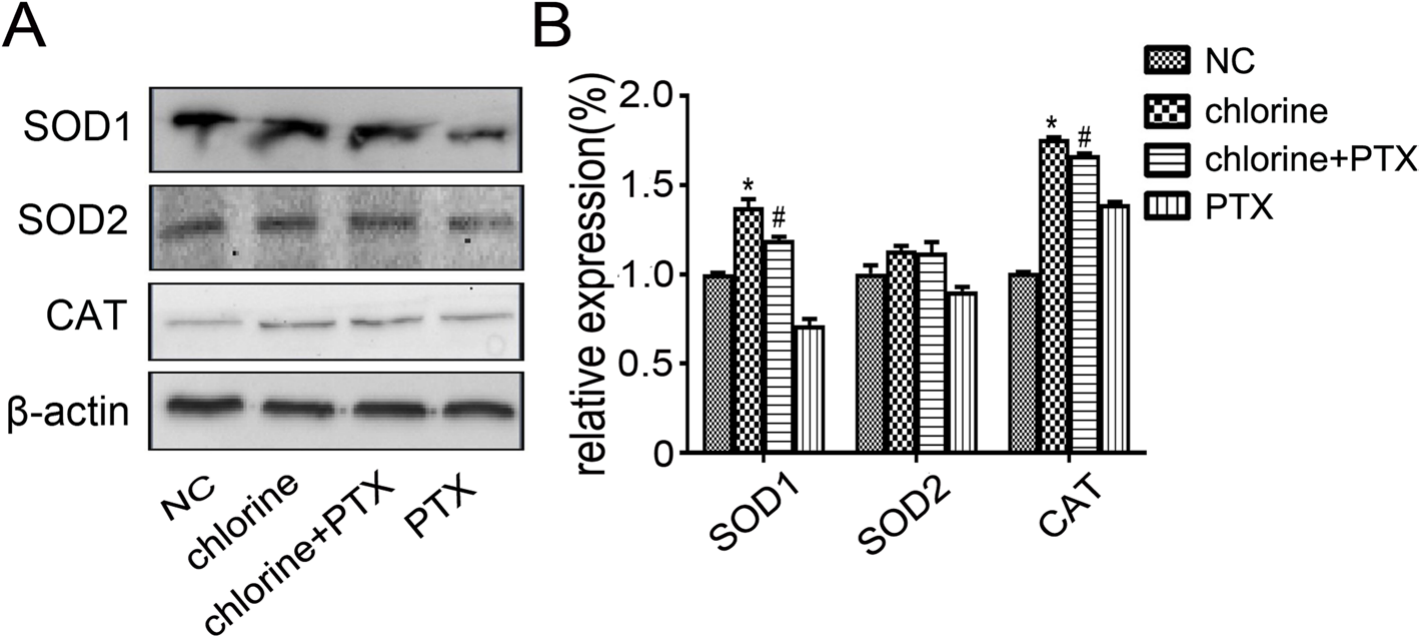
Effect of PTX on SOD1, SOD2 and CAT protein expression in rat lung tissue following chlorine exposure. **A** The protein expression levels were determined by western blot analysis. **B** Densitometric analyses of protein expression levels corresponding to (**A**). * *P* < 0.05 compared with the normal group. ^#^*P* < 0.05 compared with chlorine-treated group. NC: normal control; PTX: pentoxifylline; MDA: malondialdehyde; SOD: superoxide dismutase; CAT: catalase

Since regulation of antioxidases may be able to protect against oxidative stress, the present study further investigated whether PTX could affect the expressions of antioxidases. Therefore, the protein expressions levels of SOD 1, SOD 2 and CAT were determined. The western-blot analysis demonstrated that SOD 1 and CAT were markedly up-regulated in the chlorine-treated group compared with the NC group (*P*<0.05) (Fig. 3). Treatment with PTX could inhibit these expressions (*P*<0.05). Interestingly, chlorine did not affect the expression of SOD 2.

### Effect of PTX on the expression of LDH

**Fig. 4:**
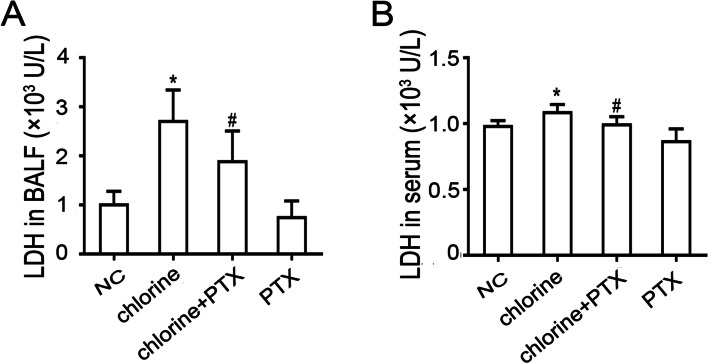
The effect of PTX on the level of LDH. The content of LDH in BALF (**A**) and serum (**B**). Data are presented as mean ± S.D. (*n* = 6). * *P* < 0.05 compared with the normal group. ^#^*P* < 0.05 compared with chlorine-treated group. NC: normal control; LDH: lactic dehydrogenase; BALF: Bronchoalveolar lavage fluid; PTX: pentoxifylline

As LDH release is positively related to cellular damage, the level of LDH was measured to calculate the degree of ALI. It was found that secretion levels of LDH both in serum and BALF were significantly increased following chlorine induction compared with the NC group, while treatments with PTX reduced increased LDH levels (*P*<0.05) (Fig. 4).

### Effect of PTX on hypoxia signaling pathway

**Fig. 5:**
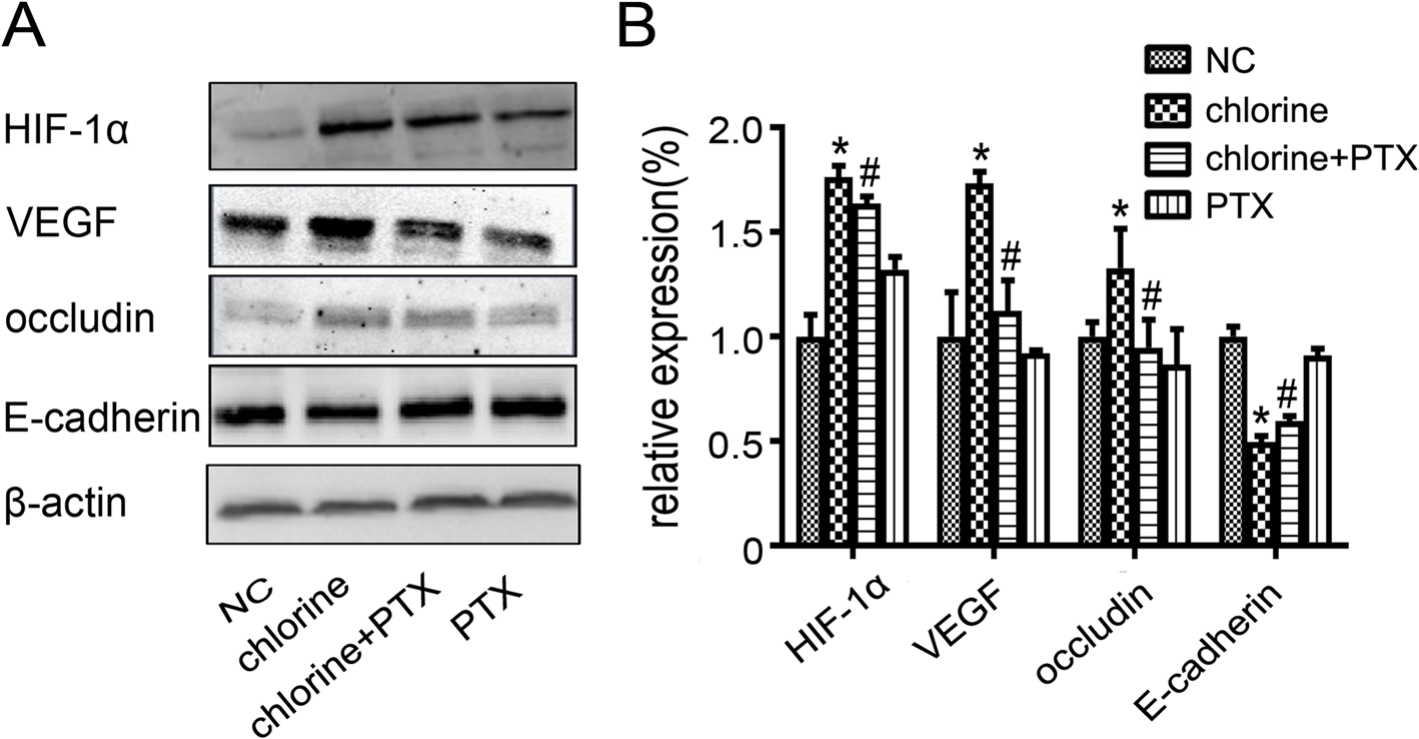
Effect of PTX on HIF-1α/VEGF signaling pathway in ALI induced by chlorine. **A** The protein expression levels were determined by western blot analysis. **B** Densitometric analyses of protein expression levels corresponding to (**A**). * *P* < 0.05 compared with the normal group. ^#^*P* < 0.05 compared with chlorine-treated group. NC: normal control; PTX: pentoxifylline; HIF-1α: Hypoxia-Inducible Factor-1α

Since hypoxemia is considered as a significant character of ALI and hypoxia activates the hypoxia signaling pathway [17], the protein expressions of HIF-1α, VEGF, occludin and E-cadherin were determined. After exposure to chlorine, expressions of HIF-1α, VEGF and occludin were significantly up-regulated in the chlorine group compared to the NC group (*P*<0.05). Administration of PTX caused a significant decrease in these indicators (*P*<0.05). In addition, the use of PTX resulted in up-regulated expression of E-cadherin (*P*<0.05), compared with the chlorine group (Fig. 5).

### Effect of PTX on autophagy

**Fig. 6:**
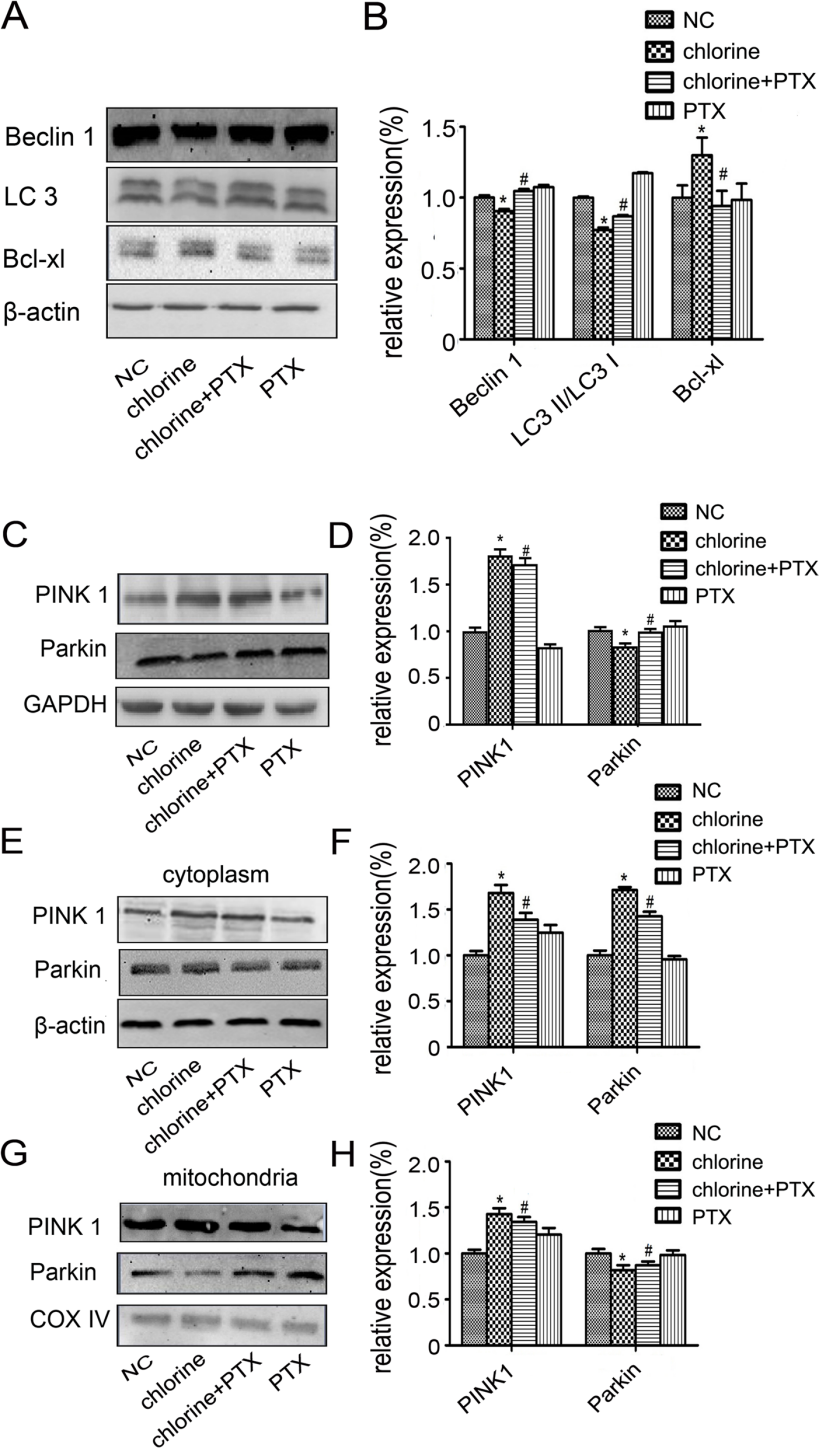
Effect of PTX on autophagy in ALI induced by chlorine. **A**, **C**, **E** and **G** The protein expression levels were determined by western blot analysis. **B**, **D**, **F** and **H** Densitometric analyses of protein expression levels corresponding to (**A**, **C**, **E** and **G**) respectively. * *P* < 0.05 compared with the normal group. ^#^*P* < 0.05 compared with chlorine-treated group. NC: normal control; PTX: pentoxifylline; PINK1: PTEN Induced Putative Kinase 1

To explore whether the protective effect of PTX was associated with autophagy, we detected the level of several key autophagy-related proteins. The results demonstrated that inhaled chlorine significantly down-regulated the ratio of LC3 II/LC3 I and the expression of Beclin-1 and increased the expression of Bcl-xl (*P*<0.05). Following treatment with PTX, the protein expression levels were obviously attenuated (*P*<0.05). To further investigate mitophagy, we searched the expressions of PINK1 and Parkin in the lung tissue. Interestingly, treatment with PTX could promote the expression of PINK1, however, inhibited the Parkin expression in the lung tissue (*P*<0.05). Because PINK1 selectively accumulates on the surface of damaged mitochondria and initiates the mitophagic process, we examined the expressions of PINK1 and Parkin in the cytoplasm and mitochondria respectively. The results showed that the PINK1 protein expression both in cytoplasm and mitochondria were significantly increased (*P*<0.05). The Parkin protein expression in cytoplasm increased while decreased in mitochondria (*P*<0.05) (Fig. 6).

## Discussion

Chlorine is a highly reactive oxidizing toxic gas which is produced globally, such as water purification, bleaching of paper, industrial manufacture of several chemicals, and for many other purposes [8]. Chlorine gas has been used as a chemical weapon since World War I. The easy availability and inherent toxicity make it attractive to aggressors willing to disrupt infrastructure or cause mass panic and casualties. Inhalation of chlorine can produce a range of acute pulmonary effects, including impaired lung function, inflammatory reactions, increase of epithelial permeability, and airway hyperresponsiveness [42]. After inhaling chlorine, the features of ALI may be epithelial cell death, inflammation, pulmonary edema, hypoxemia, and pulmonary function abnormalities, which are key aspects in animal models and human clinical studies [43]. In present chlorine-exposed rat model, we observed epithelial damage, alveolar injury and inflammation, which agreed with previous studies in several animal models [44, 45]. We also noticed pulmonary edema and ROS accumulation 3 h after chlorine exposure. These data from this study combined with our previous findings clearly suggested that rat model for chlorine-induced ALI is produced successfully.

Currently, anti-inflammatory drugs remain an effective therapy for ALI. Treatment with glucocorticoids, such as dexamethasone, led to significant improvement of lung functions and to reduced inflammation [46]. In clinical use for 30 past years, PTX has been licensed for use in peripheral vascular disease. It increases the deformability of erythrocytes, reduce blood viscosity, and inhibit fibrotic progression [47, 48]. Recent researches also demonstrated that PTX exerted beneficial effects in treating erectile dysfunction, hearing loss, Peyronie’s disease and osteoradionecrosis [49–52]. As well, in a prospective study, PTX inhibits COVID-19 severity by reduction of IL-6 and c-reactive protein (CRP) and improved prognosis of patients when combined with antioxidants [53]. In the present study, from the perspective of oxidative stress and the early research, we explored the intervention effects of PTX.

Due to oxidative stress plays a crucial role in the development of ALI [16], we measured oxidative stress markers. As an indicator of lipid peroxidation, MDA is produced in oxidative cellular damage which indicates that ROS is overproduced [54]. PTX treatment caused a significant decrease in lung tissue levels of MDA. To further investigate related mechanisms, we determined antioxidant enzymes and antioxidants. SOD is one of the major intracellular antioxidant enzymes that induces superoxide anions (O^2−^) free radical to hydrogen peroxide (H_2_O_2_). Then, H_2_O_2_ can be reduced by converting to H_2_O in the presence of CAT [55]. As one of the nature antioxidants, GSH plays important roles in reducing the tissues from damage via detoxifying electrophiles, scavenging ROS, maintaining the essential thiol status of proteins, and providing a reservoir for cysteine. During ROS formation, GSH is converted to GSH disulfide (GSSG). In the current study, PTX also suppressed the levels of SOD1, GSSG and expression of CAT, and enhanced the GSH level and GSH/GSSG ratio to protect against pulmonary injury. However, there appeared no obvious effects on SOD activity when treated with PTX. By detecting the protein expressions of SOD1 and SOD2, we found that the expression level of SOD2 protein showed no significant difference between groups. Thus, the failure of PTX to reverse SOD activity may be related to the stable expression of SOD2. Based on the observation, we suggested that PTX has a beneficial antioxidative effect on ALI induced by chlorine. Since the activity level of LDH reflects the degree of cell injury, subsequently, we selected it as a functional indicator of ALI. The results showed that PTX treatment ameliorated the level of the LDH both in BALF and serum effectively, indicating the protective effect of PTX in lung injury induced by chlorine.

Hypoxia is closely related to oxidative stress in inflammatory lung diseases [56]. In response to hypoxia, HIF-1α binds to the hypoxia response element of the erythropoietin gene and controls the hypoxic induction of HIF-1-mediated gene transcription. In addition, as a transcriptional heterodimer, HIF-1exerts a vital pathophysiological role in oxygen homeostasis [57]. Jahani et al. considered that hypoxia might be a key feature of COVID-19 launching activation of HIF-1 [58]. Under hypoxic conditions, ROS released from the mitochondrial electron transport chain can participate in the regulation of HIF-1 activity [59]. In this study, we showed that PTX directly reversed overexpression of HIF-1α. The classical HIF-1α/VEGF signaling pathway also exerts an important role in the pathogeneses of ALI and pulmonary edema. Besides, HIF-1α induces and activates the overexpression of the VEGF gene, which consequently affects the expression of tight junction proteins and adhesion molecules [60, 61]. The present study found that PTX significantly inhibited the overexpression of VEGF and occludin accompanied by the upregulation of E-cadherin, which were in agreement with previous researches [62, 63]. These findings showed that the ROS/HIF-1α/VEGF signaling pathway in the lung tissues of rat models in chlorine-induced ALI was activated.

Autophagy, one type of cell death, is a mechanism for cell self-protection and self-renewal which relies on lysosomes to degrade their own organelles or proteins. As a major cellular defense against oxidative stress, autophagy is an intracellular digestion system that works as an inducible adaptive response to ALI. ROS may activate autophagy, and then facilitate cellular adaptation and diminish the damaged macromolecules and dysfunctional organelles [64]. However, the role of autophagy in the mechanism of ALI has been controversial. In a diabetic rat model, when treated with autophagy inhibitor 3-methyladenine, the results showed more serious ALI [65]. Numerous regulators like LC3 II and Beclin 1 play important role in process of autophagy induction during lung injury. After binding to the lipid derivative phosphatidylethanolamine, LC 3 I is converted to form LC 3 II, which enables fusion with the lysosomes. In addition, the ratio of LC 3 I/LC 3 II is used as an indicator of autophagy. As a part of a Class III PI3K complex, Beclin 1 takes part in autophagosome formation though assembling around cargo in a vesicle and combining with lysosome [66]. As our results demonstrated, PTX enhanced the expression of LC3 II and Beclin 1 accompanied by the reducing of Bcl-xl, suggesting that autophagy exerted a protective role in ALI induced by chlorine.

Because mitochondria are considered as the main contributor of reactive oxygen species, the removal of damaged mitochondria by mitophagy plays important role in cellular antioxidant defenses [67]. PINK1, as a mitochondrially targeted serine–threonine kinase, takes part in mitochondrial quality control. Under normal conditions, PINK 1 maintains low basal levels though importing into the mitochondrial intermembrane space and rapidly degraded when combined with the presenilin-associated rhomboid-like protein (PARL) and the proteasome. When mitochondria are depolarization, PINK1 accumulates on the mitochondrial outer membrane (OMM) and results in recruitment of Parkin from the cytosol, then activates mitophagy [68]. Subsequently, the expressions of PINK1 and Parkin were performed to investigate the potential mechanism. Interestingly, PTX had been shown to inhibit the expression of PINK1, but increased the expression of Parkin. After separating mitochondria and cytoplasm, we found that the expression of PINK1 and Parkin in mitochondria showed similar trends with these expression in lung tissues. However, PTX treatment reduced the expression of PINK1 and Parkin in cytoplasm compared to the chlorine group. These results showed that PTX exerted a protective role in attenuating ALI induced by chlorine through improving autophagy, especially mitophagy.

In conclusion, the present research demonstrated that PTX might attenuate chlorine induced ALI through regulating oxidative stress, hypoxia and autophagy. However, more research will be needed to explore specific mechanism, which is our direction in the future.

## Supplementary Information

**Additional file 1.**

## Data Availability

The datasets used and/or analyzed during the current study are available from the corresponding author on reasonable request.

## References

[1] Yadav AK, Bracher A, Doran SF, Leustik M, Squadrito GL (2010). Mechanisms and modification of chlorine-induced lung injury in animals. Proc Am Thorac Soc.

[2] Honavar J, Doran S, Ricart K, Matalon S, Patel RP (2017). Nitrite therapy prevents chlorine gas toxicity in rabbits. Toxicol Lett.

[3] Zhang B, Shen H, Yun X, Zhong QR, Henderson BH, Wang X (2022). Global emissions of hydrogen chloride and particulate chloride from continental sources. Environ Sci Technol.

[4] Jones R, Wills B, Kang C (2010). Chlorine gas: an evolving hazardous material threat and unconventional weapon. West J Emerg Med.

[5] Winder C (2001). The toxicology of chlorine. Environ Res.

[6] Szinicz L (2005). History of chemical and biological warfare agents. Toxicology..

[7] Jacobs D, Kovac A (2020). The introduction of gas warfare and its medical response in world war one. J Anesth Hist.

[8] Achanta S, Jordt SE (2021). Toxic effects of chlorine gas and potential treatments: a literature review. Toxicol Mech Methods.

[9] Sexton JD, Pronchik DJ (1998). Chlorine inhalation: the big picture. J Toxicol Clin Toxicol.

[10] Gorguner M, Aslan S, Inandi T, Cakir Z (2004). Reactive airways dysfunction syndrome in housewives due to a bleach-hydrochloric acid mixture. Inal Toxicol.

[11] Liu SS, Qu HM, Yang D, Hu H, Liu WL, Qiu ZG (2018). Chlorine disinfection increases both intracellular and extracellular antibiotic resistance genes in a full-scale wastewater treatment plant. Water Res.

[12] Chauhan S, Chauhan S, D'Cruz R, Faruqi S, Singh KK, Varma S (2008). Chemical warfare agents. Environ Toxicol Pharmacol.

[13] Cho YJ, Moon JY, Shin ES, Kim JH, Jung H, Park SY (2016). Clinical practice guideline of acute respiratory distress syndrome. Tuberc Respir Dis (Seoul).

[14] Lang JD, McArdle PJ, O'Reilly PJ, Matalon S (2002). Oxidant-antioxidant balance in acute lung injury. Chest..

[15] Kellner M, Noonepalle S, Lu Q, Srivastava A, Zemskov E, Black SM (2017). ROS signaling in the pathogenesis of acute lung injury (ALI) and acute respiratory distress syndrome (ARDS). Adv Exp Med Biol.

[16] Duan Q, Jia Y, Qin Y, Jin Y, Hu H, Chen J (2020). Narciclasine attenuates LPS-induced acute lung injury in neonatal rats through suppressing inflammation and oxidative stress. Bioengineered..

[17] Lee JW, Ko J, Ju C, Eltzschig HK (2019). Hypoxia signaling in human diseases and therapeutic targets. Exp Mol Med.

[18] Semenza GL, Wang GL (1992). A nuclear factor induced by hypoxia via de novo protein synthesis binds to the human erythropoietin gene enhancer at a site required for transcriptional activation. Mol Cell Biol.

[19] Li HS, Zhou YN, Li L, Long D, Chen XL, Zhang JB (2019). HIF-1α protects against oxidative stress by directly targeting mitochondria. Redox Biol.

[20] Wei XX, Chen YH, Jiang XJ, Peng M, Liu YD, Mo YZ (2021). Mechanisms of vasculogenic mimicry in hypoxic tumor microenvironments. Mol Cancer.

[21] Zhang XD, Yu WH, Liu MM, Liu R, Wu H, Wang Z, et al. Pentoxifylline inhibits phosgene-induced lung injury via improving hypoxia. Drug Chem Toxicol. 2022:1–8. 10.1080/01480545.2022.2131811. 10.1080/01480545.2022.2131811 36220803

[22] Zhang XD, Hai CX, Cai FL, Liang X, Liu R, Chen HL (2008). Time course for expression of VEGF and its receptor and regulator levels of contraction and relaxation in increased vascular permeability of lung induced by phosgene. Inhal Toxicol.

[23] Li X, Shan C, Wu Z, Yu H, Yang A, Tan B (2020). Emodin alleviated pulmonary inflammation in rats with LPS-induced acute lung injury through inhibiting the mTOR/HIF-1alpha/VEGF signaling pathway. Inflamm Res.

[24] Sharada P, Swaminathan U, Nagamalini BR, Kumar KV, Ashwini BK, Lavanya V (2018). Coalition of E-cadherin and vascular endothelial growth factor expression in predicting malignant transformation in common oral potentially malignant disorders. J Oral Maxillofac Pathol.

[25] Ghosh B, Loube J, Thapa S, Ryan H, Capodanno E, Chen D (2022). Loss of E-cadherin is causal to pathologic changes in chronic lung disease. Commun Biol.

[26] Yu L, Chen Y, Tooze SA (2018). Autophagy pathway: cellular and molecular mechanisms. Autophagy..

[27] Liao SX, Sun PP, Gu YH, Rao XM, Zhang LY, Ou-Yang Y (2019). Autophagy and pulmonary disease. Ther Adv Respir Dis.

[28] Lin L, Zhang L, Yu L, Han L, Ji W, Shen H, Hu Z (2016). Time-dependent changes of autophagy and apoptosis in lipopolysaccharide-induced rat acute lung injury. Iran J Basic Med Sci.

[29] Zhao H, Chen H, Xiaoyin M, Yang G, Hu Y, Xie K (2019). Autophagy activation improves lung injury and inflammation in sepsis. Inflammation..

[30] Zhang RH, Zhang HL, Li PY, Li CH, Gao JP, Li J (2021). Autophagy is involved in the replication of H9N2 influenza virus via the regulation of oxidative stress in alveolar epithelial cells. Virol J.

[31] Ward A, Clissold SP (1987). Pentoxifylline. A review of its pharmacodynamic and pharmacokinetic properties, and its therapeutic efficacy. Drugs..

[32] Li H, Tan G, Tong L, Han P, Zhang F, Liu B, Sun X (2016). Pentoxifylline inhibits pulmonary inflammation induced by infrarenal aorticcross-clamping dependent of adenosine receptor A2A. Am J Transl Res.

[33] Bektas S, Karakaya K, Can M, Bahadir B, Guven B, Erdogan N (2016). The effects of tadalafil and pentoxifylline on apoptosis and nitric oxide synthase in liver ischemia/reperfusion injury. Kaohsiung J Med Sci.

[34] Yao C, Li G, Qian Y, Cai M, Yin H, Xiao L (2016). Protection of pentoxifylline against testis injury induced by intermittent hypobaric hypoxia. Oxid Med Cell Longev.

[35] Kudoh I, Ohtake M, Nishizawa H, Kurahashi K, Hattori S, Okumura F (1995). The effect of pentoxifylline on acid-induced alveolar epithelial injury. Anesthesiology..

[36] Kudoh I, Miyazaki H, Ohara M, Fukushima J, Tazawa T, Yamada H (2001). Activation of alveolar macrophages in acid-injured lung in rats: different effects of pentoxifylline on tumor necrosis factor-alpha and nitric oxide production. Crit Care Med.

[37] Welsh CH, Lien D, Worthen GS, Weil JV (1988). Pentoxifylline decreases endotoxin-induced pulmonary neutrophil sequestration and extravascular protein accumulation in the dog. Am Rev Respir Dis.

[38] Michetti C, Coimbra R, Hoyt DB, Loomis W, Junger W, Wolf P (2003). Pentoxifylline reduces acute lung injury in chronic endotoxemia. J Surg Res.

[39] Mostafa-Hedeab G, Al-Kuraishy HM, Al-Gareeb AI, Jeandet P, Saad HM, Batiha GE (2022). A raising dawn of pentoxifylline in management of inflammatory disorders in Covid-19. Inflammopharmacology..

[40] Liu ML, Dong HY, Zhang B, Zheng WS, Zhao PT, Liu Y (2012). Insulin reduces LPS-induced lethality and lung injury in rats. Pulm Pharmacol Ther.

[41] Guo P, Li B, Liu MM, Li YX, Weng GY, Gao Y (2022). Protective effects of lotus plumule ethanol extracts on bleomycin-induced pulmonary fibrosis in mice. Drug Chem Toxicol.

[42] Elfsmark L, Agren L, Akfur C, Bucht A, Jonasson S (2018). 8-Isoprostane is an early biomarker for oxidative stress in chlorine-induced acute lung injury. Toxicol Lett.

[43] Musah S, Schlueter CF, Humphrey DJ, Powell KS, Roberts AM, Hoyle GW (2017). Acute lung injury and persistent small airway disease in a rabbit model of chlorine inhalation. Toxicol Appl Pharmacol.

[44] Hoyle GW, Svendsen ER (2016). Persistent effects of chlorine inhalation on respiratory health. Ann N Y Acad Sci.

[45] Watkins R, Perrott R, Bate S, Auton P, Watts S, Stoll A (2021). Development of chlorine-induced lung injury in the anesthetized, spontaneously breathing pig. Toxicol Mech Methods.

[46] Mikolka P, Kosutova P, Kolomaznik M, Topercerova J, Kopincova J, Calkovska A (2019). Effect of different dosages of dexamethasone therapy on lung function and inflammation in an early phase of acute respiratory distress syndrome model. Physiol Res.

[47] Feng C, Zhang M, Zhang S, Zhang J, Li C, Zhou J (2021). Therapeutic effects of pentoxifylline on invasive pulmonary aspergillosis in immunosuppressed mice. BMC Pulm Med.

[48] González-Pacheco H, Amezcua-Guerra LM, Sandoval J, Arias-Mendoza A (2020). Potential usefulness of pentoxifylline, a non-specific phosphodiesterase inhibitor with anti-inflammatory, anti-thrombotic, antioxidant, and anti-fibrogenic properties, in the treatment of SARS-CoV-2. Eur Rev Med Pharmacol Sci.

[49] Lu Y, Su H, Zhang JZ, Wang YT, Li HJ (2021). Treatment of poor sperm quality and erectile dysfunction with oral pentoxifylline: a systematic review. Front Pharmacol.

[50] Lan WC, Wang C, Lin CD (2018). Pentoxifylline versus steroid therapy for idiopathic sudden sensorineural hearing loss with diabetes. J Int Adv Otol.

[51] Ibrahim A, Gazzard L, Alharbi M, Rompré-Brodeur A, Aube M, Carrier S (2019). Evaluation of oral pentoxifylline, colchicine, and penile traction for the management of Peyronie's disease. Sex Med.

[52] Dissard APDN, Barthelemy I, Delbet C, Puechmaille M, Depeyre A (2020). Efficacy of pentoxifylline-tocopherol-clodronate in mandibular osteoradionecrosis. Laryngoscope..

[53] Chavarria AP, Vazquez R, Cherit JD, Bello HH, Suastegui HC, Moreno-Castañeda L (2021). Antioxidants and pentoxifylline as coadjuvant measures to standard therapy to improve prognosis of patients with pneumonia by COVID-19. Comput Struct Biotechnol J.

[54] Hajam YA, Rani R, Ganie SY, Sheikh TA, Javaid D, Qadri SS (2022). Oxidative stress in human pathology and aging: molecular mechanisms and perspectives. Cells..

[55] Zhang J, Duan D, Song ZL, Liu T, Hou Y, Fang J (2021). Small molecules regulating reactive oxygen species homeostasis for cancer therapy. Med Res Rev.

[56] Xiong M, Zhao Y, Mo H, Yang H, Yue F, Hu K (2021). Intermittent hypoxia increases ROS/HIF-1alpha ‘Related oxidative stress and inflammation and worsens bleomycin-induced pulmonary fibrosis in adult male C57BL/6J mice. Int Immunopharmacol.

[57] Liu Y, Xiang D, Zhang H, Yao H, Wang Y (2020). Hypoxia-inducible factor-1: a potential target to treat acute lung injury. Oxid Med Cell Longev.

[58] Jahani M, Dokaneheifard S, Mansouri K (2020). Hypoxia: a key feature of COVID-19 launching activation of HIF-1 and cytokine storm. J Inflamm (Lond).

[59] Horak P, Crawford AR, Vadysirisack DD, Nash ZM, DeYoung MP, Sgroi D (2010). Negative feedback control of hIF-1 through REDD1-regulated ROS suppresses tumorigenesis. Proc Natl Acad Sci U S A.

[60] McClendon J, Jansing NL, Redente EF, Gandjeva A, Ito Y, Colgan SP (2017). Hypoxia-inducible factor 1Alpha signaling promotes repair of the alveolar epithelium after acute lung injury. Am J Pathol.

[61] Li M, Li G, Yu B, Luo Y, Li Q (2020). Activation of hypoxia-inducible factor-1alpha via succinate dehydrogenase pathway during acute lung injury induced by trauma/hemorrhagic shock. Shock..

[62] Nathan JR, Lakshmanan G, Michael FM, Seppan P, Ragunathan M (2016). Expression of adenosine receptors and Vegf during angiogenesis and its inhibition by pentoxifylline-a study using zebrafish model. Biomed Pharmacother.

[63] Costantini TW, Loomis WH, Putnam JG, Kroll L, Eliceiri BP, Baird A (2009). Pentoxifylline modulates intestinal tight junction signaling after burn injury: effects on myosin light chain kinase. J Trauma.

[64] Ornatowski W, Lu Q, Yegambaram M, Garcia AE, Zemskov EA (2020). Complex interplay between autophagy and oxidative stress in the development of pulmonary disease. Redox Biol.

[65] Zhan L, Zhang Y, Su W, Zhang Q, Chen R, Zhao B (2018). The roles of autophagy in acute lung injury induced by myocardial ischemia reperfusion in diabetic rats. J Diabetes Res.

[66] Vishnupriya S, Priya DL, Sakthivel KM, Rasmi RR (2020). Autophagy markers as mediators of lung injury-implication for therapeutic intervention. Life Sci.

[67] Zhang Y, Wong HS (2021). Are mitochondria the main contributor of reactive oxygen species in cells?. J Exp Biol.

[68] Hamacher-Brady A, Brady NR (2016). Mitophagy programs: mechanisms and physiological implications of mitochondrial targeting by autophagy. Cell Mol Life Sci.

