# Supplementary figures and images for: Protective effects of pentoxifylline against chlorine-induced acute lung injury in rats
Source: BMC Pharmacol Toxicol. 2023 Feb 27;24:12. doi: 10.1186/s40360-023-00645-2 (PMC9969370; doi:10.1186/s40360-023-00645-2)

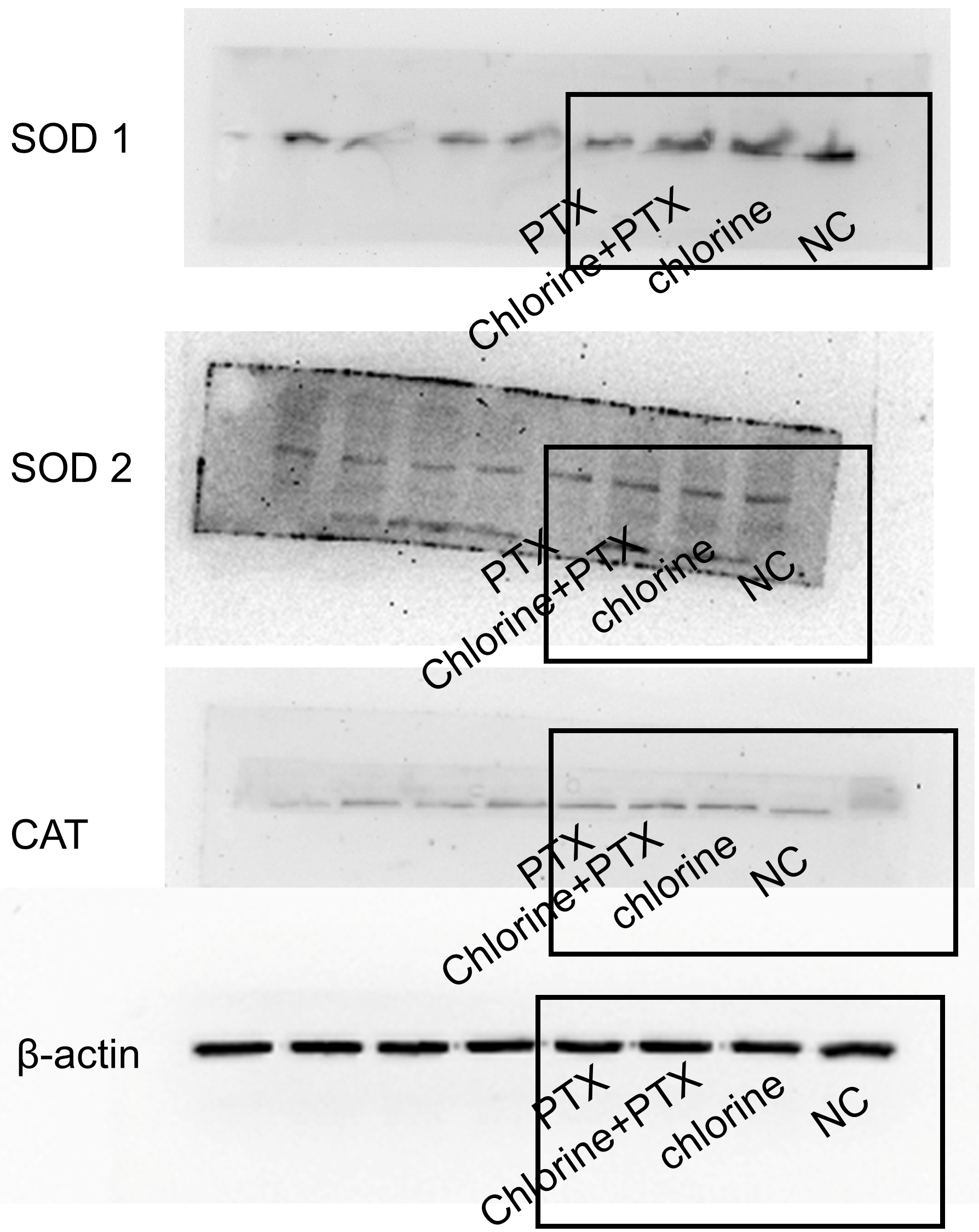

Supplement: Supplementary file 1 — Additional file 1. [file 40360_2023_645_MOESM1_ESM.zip › Figure S1.TIF]

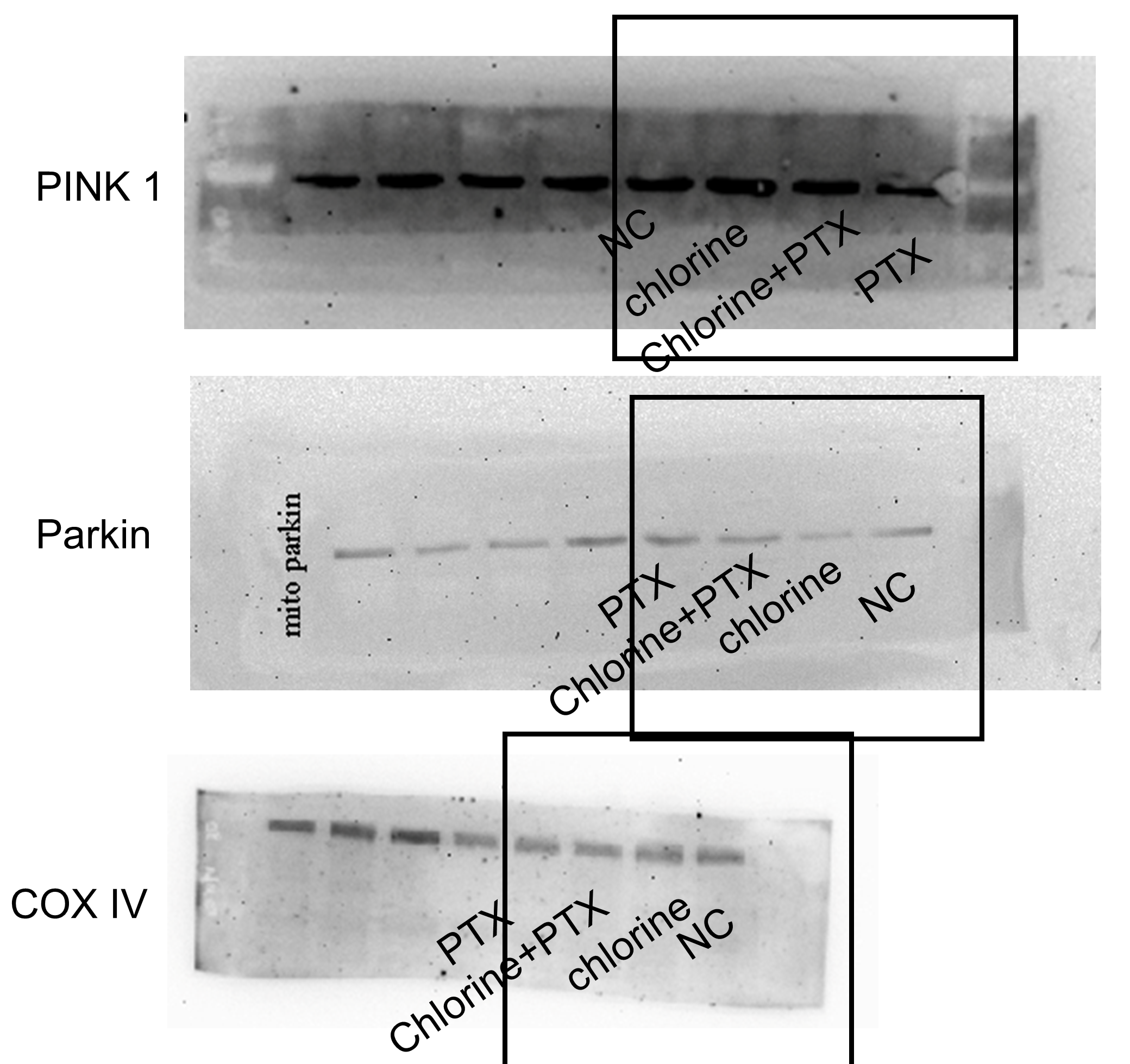

Supplement: Supplementary file 1 — Additional file 1. [file 40360_2023_645_MOESM1_ESM.zip › Figure S10.TIF]

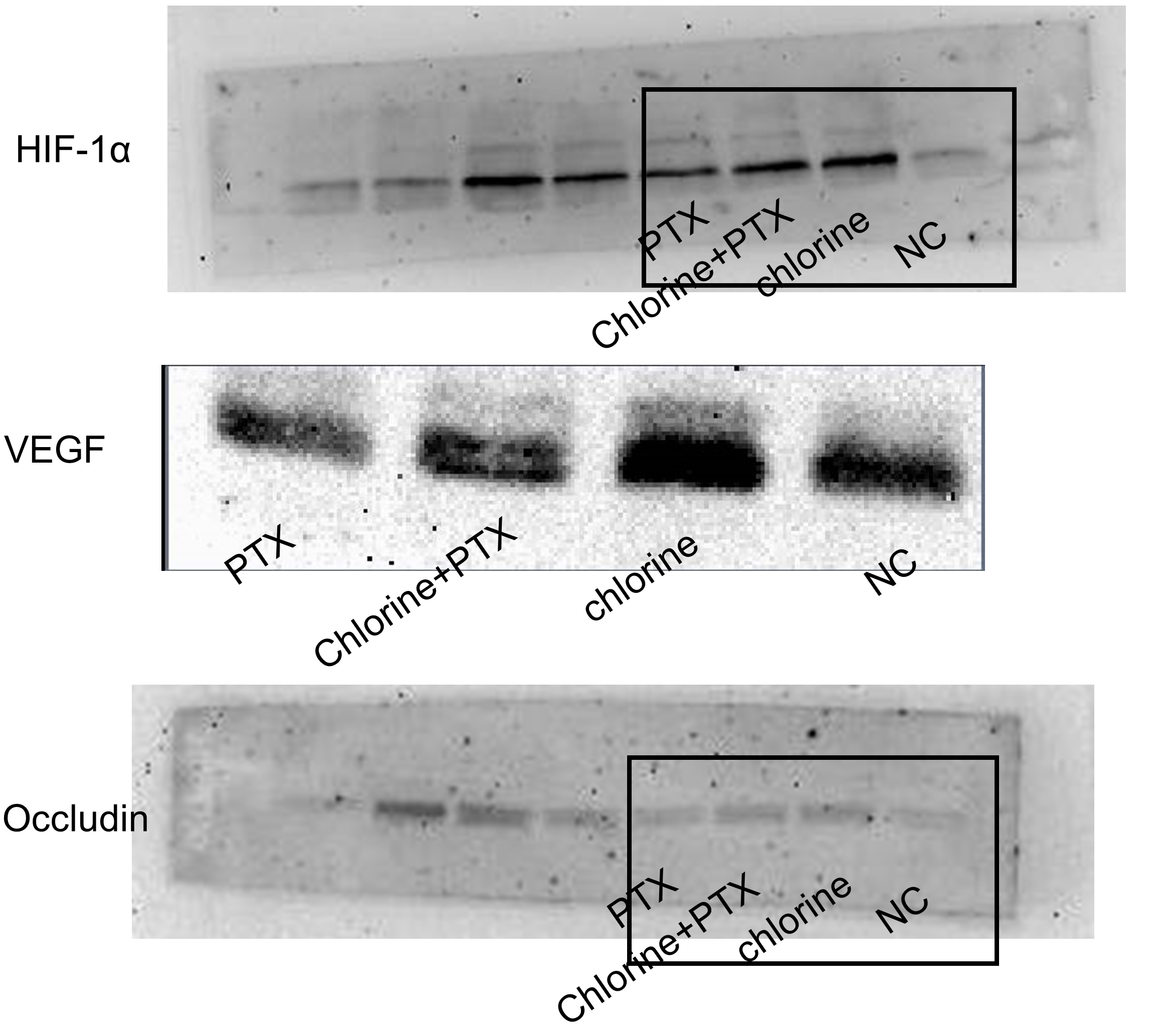

Supplement: Supplementary file 1 — Additional file 1. [file 40360_2023_645_MOESM1_ESM.zip › Figure S2.TIF]

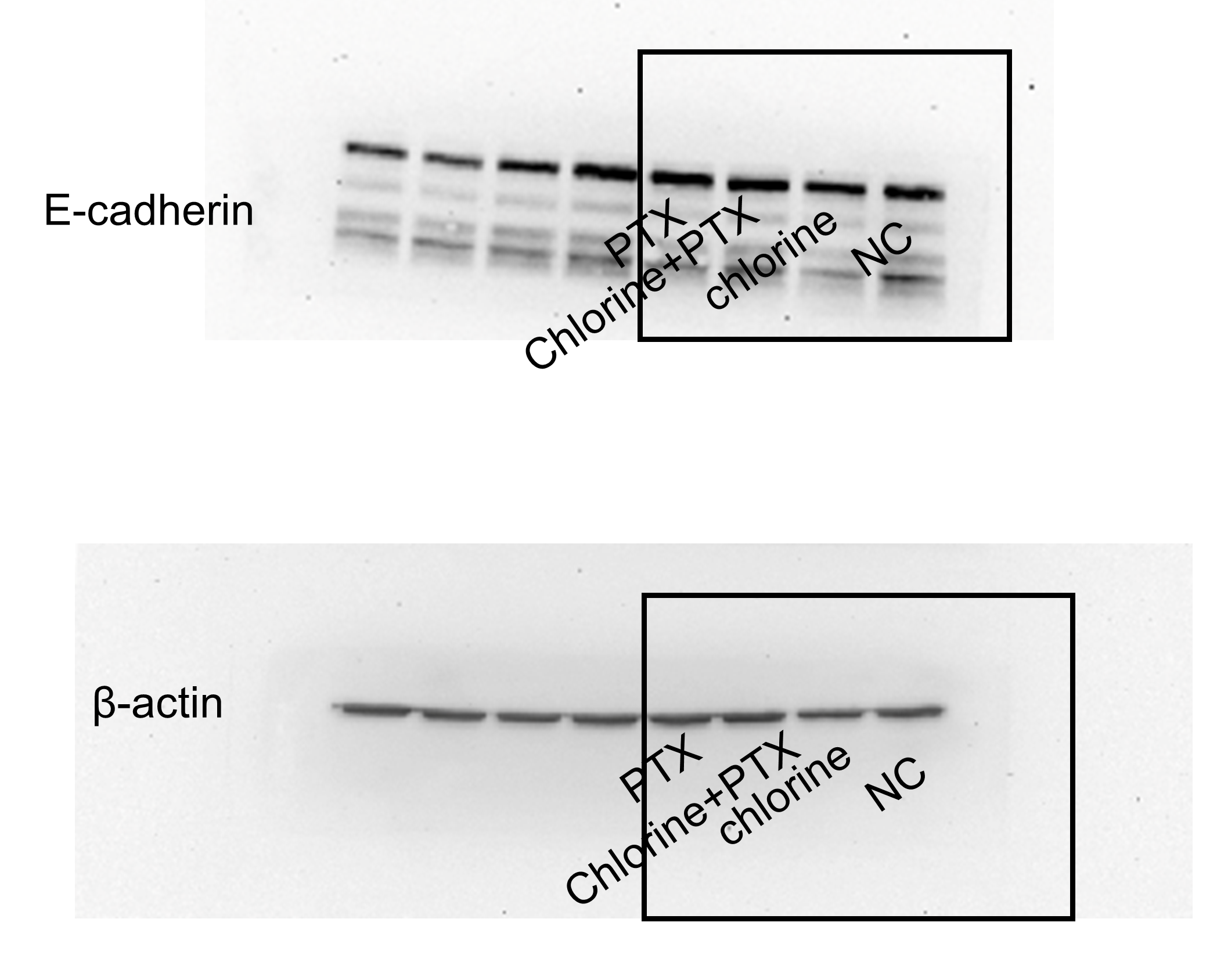

Supplement: Supplementary file 1 — Additional file 1. [file 40360_2023_645_MOESM1_ESM.zip › Figure S3.TIF]

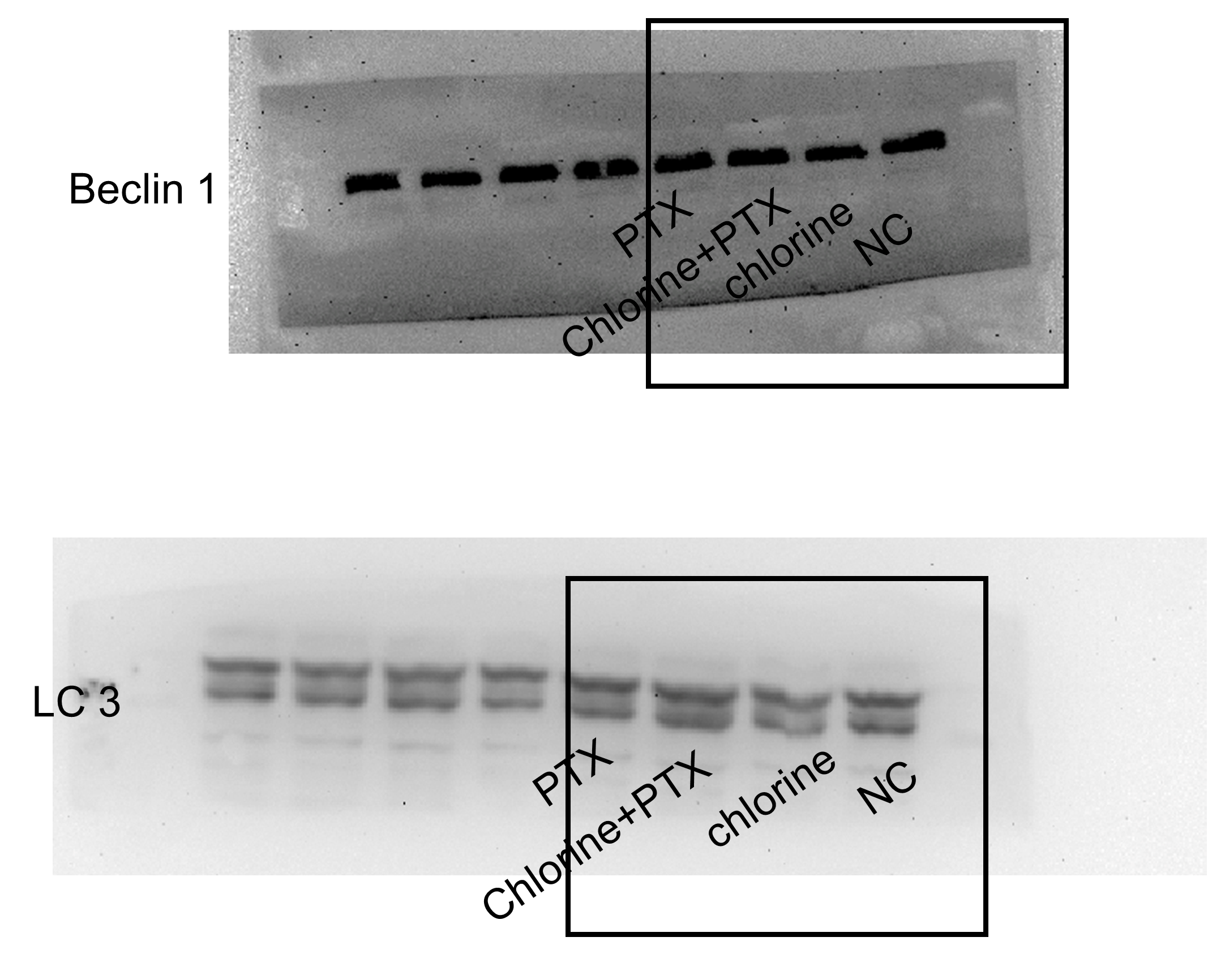

Supplement: Supplementary file 1 — Additional file 1. [file 40360_2023_645_MOESM1_ESM.zip › Figure S4.TIF]

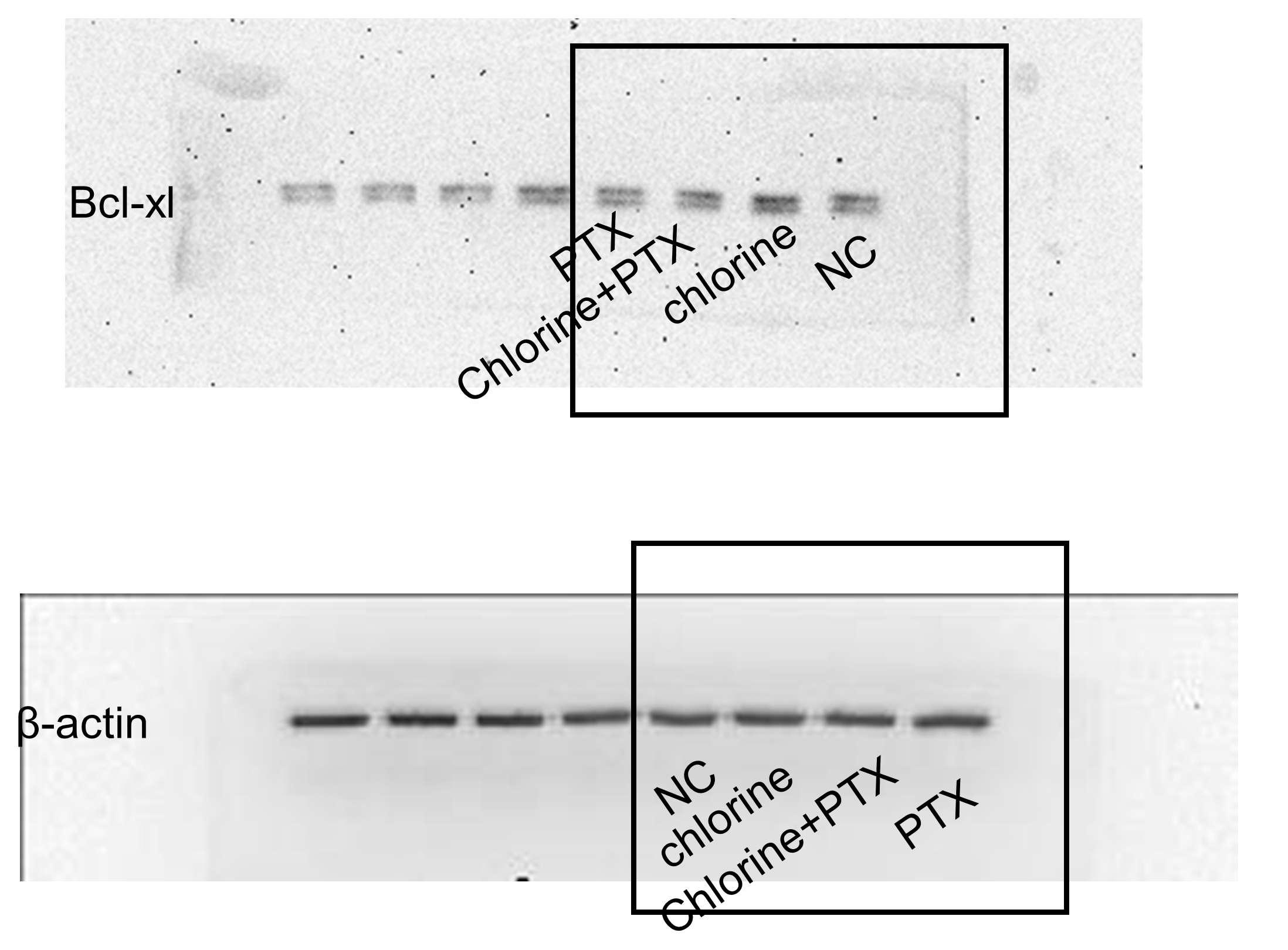

Supplement: Supplementary file 1 — Additional file 1. [file 40360_2023_645_MOESM1_ESM.zip › Figure S5.TIF]

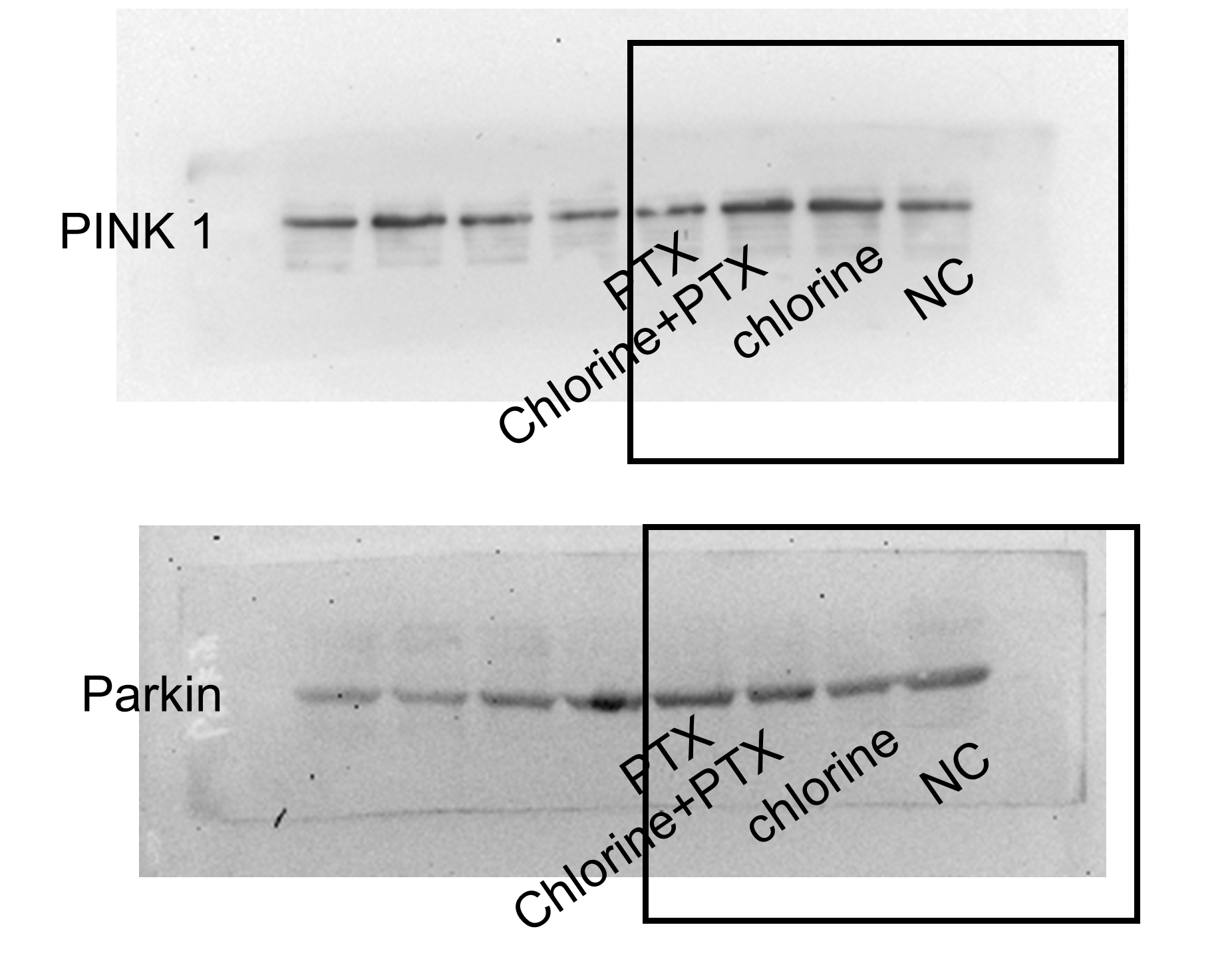

Supplement: Supplementary file 1 — Additional file 1. [file 40360_2023_645_MOESM1_ESM.zip › Figure S6.TIF]

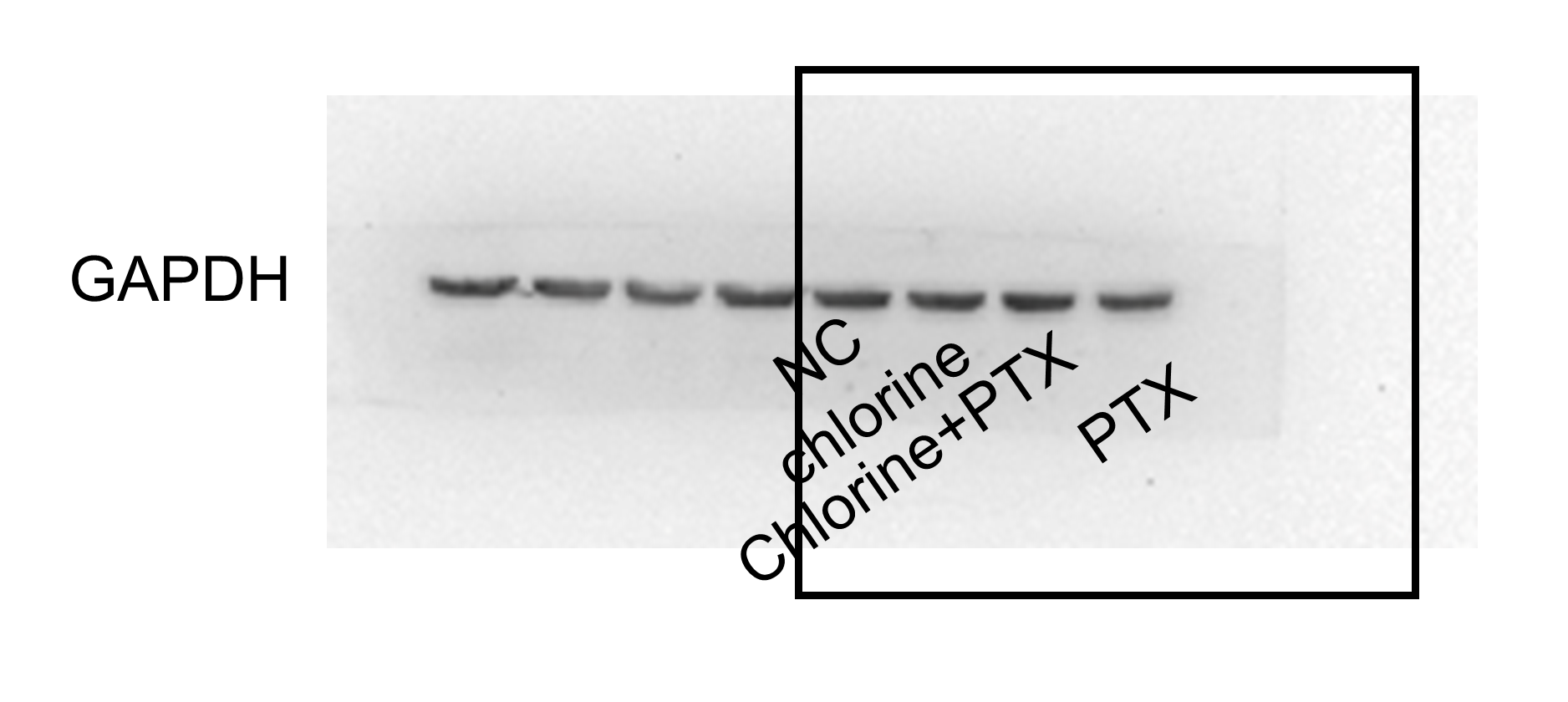

Supplement: Supplementary file 1 — Additional file 1. [file 40360_2023_645_MOESM1_ESM.zip › Figure S7.TIF]

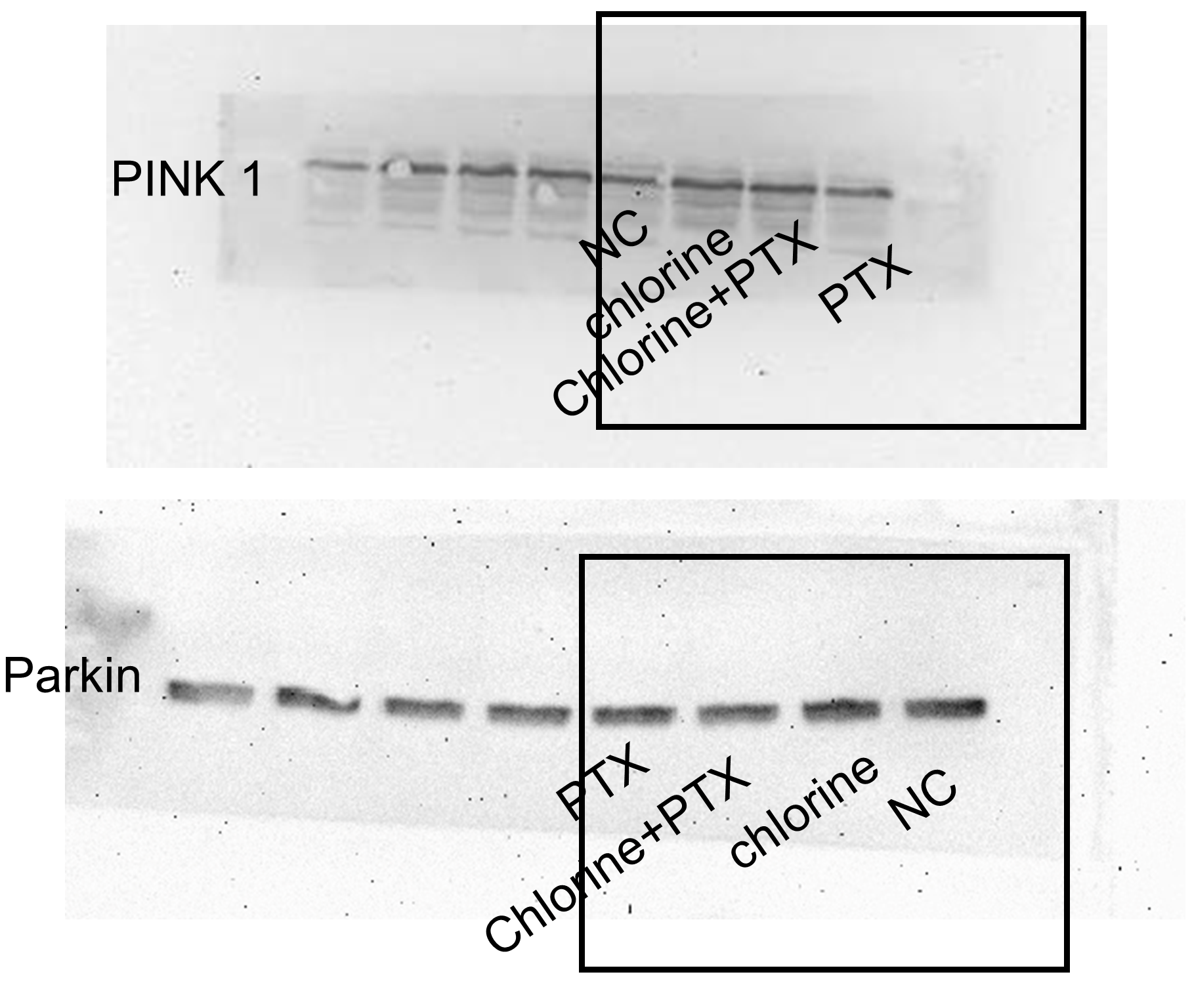

Supplement: Supplementary file 1 — Additional file 1. [file 40360_2023_645_MOESM1_ESM.zip › Figure S8.TIF]

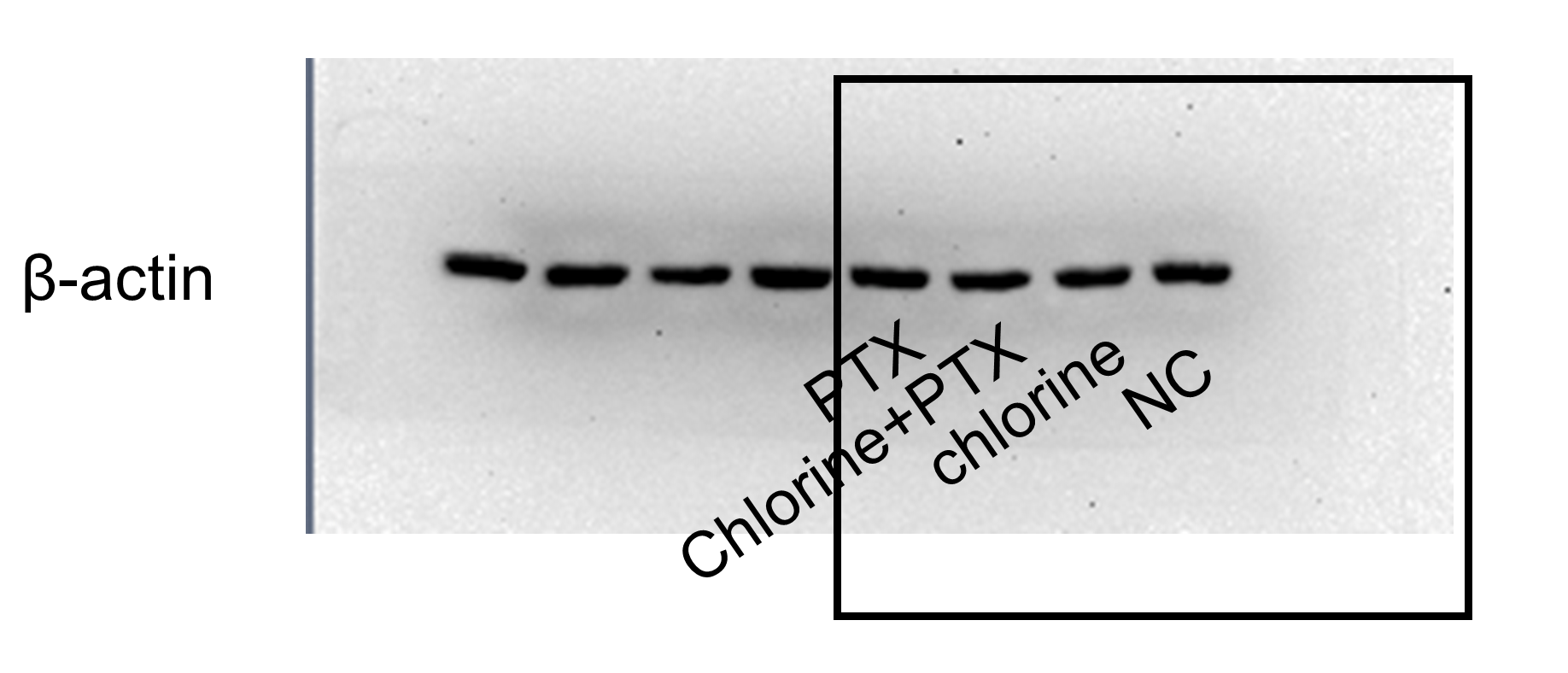

Supplement: Supplementary file 1 — Additional file 1. [file 40360_2023_645_MOESM1_ESM.zip › Figure S9.TIF]
